# The *Escherichia coli* O157:H7 carbon starvation-inducible lipoprotein Slp contributes to initial adherence *in vitro* via the human polymeric immunoglobulin receptor

**DOI:** 10.1371/journal.pone.0216791

**Published:** 2019-06-12

**Authors:** Christine Fedorchuk, Indira T. Kudva, Subhashinie Kariyawasam

**Affiliations:** 1 Department of Veterinary and Biomedical Science, The Pennsylvania State University, University Park, Pennsylvania, United States of America; 2 Food Safety and Enteric Pathogens Research Unit, National Animal Disease Center, Agricultural Research Service, U S Department of Agriculture, Ames, Iowa, United States of America; 3 Department of Comparative, Diagnostic, and Population Medicine, College of Veterinary Medicine, University of Florida, Gainesville, Florida, United States of America; USDA-ARS Eastern Regional Research Center, UNITED STATES

## Abstract

*Escherichia coli* O157:H7 is the most well-studied serotype of the enterohemorrhagic *E*. *coli* (EHEC) class of *E*. *coli* intestinal pathogens and is responsible for many outbreaks of serious food-borne illness worldwide each year. Adherence mechanisms are a critical component of its pathogenesis, persistence in natural reservoirs, and environmental contamination. *E*. *coli* O157:H7 has a highly effective virulence operon, the Locus of Enterocyte Effacement (LEE), and its encoded intimate adherence mechanism is well characterized. However, factors involved in the preceding initial attachment are not well understood. In this study, we propose a mechanism of initial adherence used by *E*. *coli* O157:H7 *in vitro*. We describe a bacterial protein not previously reported to be involved in adherence, Slp, and its interactions with the human host protein polymeric immunoglobulin receptor (pIgR). The human pIgR has previously been shown to act as an adherence receptor for some mucosal pathogens and is highly expressed in the intestine. Following observation of significant colocalization between *E*. *coli* O157:H7 bacteria and pIgR location on Caco-2 cells, a co-immunoprecipitation (Co-IP) assay using a human recombinant Fc-tagged pIgR protein led to the identification of this protein. Disruption of Slp expression in *E*. *coli* O157:H7, through deletion of its encoding gene *slp*, produced a significant adherence deficiency to Caco-2 cells at early time points associated with initial adherence. Plasmid complementation of the *slp* gene fully restored the wild-type phenotype. Furthermore, immunofluorescence microscopy revealed evidence that this interaction is specific to the pathogenic strains of *E*. *coli* tested and not the nonpathogenic control strain *E*. *coli* K12. Additionally, deletion of *slp* gene resulted in the absence of the corresponding protein band in further Co-IP assays, while the plasmid-encoded *slp* gene complementation of the deletion mutant strain restored the wild-type pattern. These data support the proposal that Slp directly contributes to initial adherence, with the pIgR protein as its proposed receptor.

## Introduction

It has been estimated that over 700 outbreaks of foodborne illness due to *Escherichia coli* occurred in the United States between 2007 and 2017 [1]. Of these reported outbreaks, over 99% were due to Enterohemorrhagic *E*. *coli* (EHEC) strains, with approximately 65% belonging to the O157 serogroup [1]. EHEC infections typically result in abdominal cramping, watery or bloody diarrhea, fever, nausea, and vomiting [2]. Among these cases, a subset develop a severe complication known as hemolytic uremic syndrome (HUS), which is primarily observed in infected children under the age of five years, the elderly, and immunocompromised individuals [3]. In the U.S., *E*. *coli* O157:H7 outbreaks have highly variable rates of HUS ranging from less than 1% to greater than 15% [4, 5].

After ingestion from a contaminated source, *E*. *coli* O157:H7 uses a series of adherence mechanisms to colonize the intestine during infection. Adherence occurs in two phases: (i) initial adherence (an early, loose attachment) and (ii) intimate adherence (strong attachment) [6]. Upon infiltration of the mucin layer in the colon, the bacteria require an initial attachment in order to stay stably adjacent to the intestinal epithelial cell (IEC). The elapsed time must be sufficient for the opportunity to engage its intimate adherence mechanism before being cleared from the digestive tract [7]. *E*. *coli* O157:H7 utilizes a fairly well-characterized set of virulence factors for intimate adherence, using factors encoded in the Locus of Enterocyte Effacement (LEE) [8]. The three main factors required for intimate adherence are (i) intimin, (ii) Tir, and (iii) type three secretion system (T3SS), which are the bacterial adhesin, translocated intimin receptor, and secretion apparatus, respectively [9]. An additional receptor for intimin, the host protein nucleolin (involved in cell proliferation and growth), has also been shown to assist in intimate adherence [10, 11].

The earlier phase of initial adherence is less well defined, and it is likely that initial adherence is governed by multiple redundant mechanisms and varies between strains [6]. Several putative adhesins have been described, but the specific contribution to initial adherence is unclear. For example, long polar fimbriae (LPF) have recently been described as initial adhesins in *E*. *coli* O157:H7, but with some conflicting reports. LPF has been described in the role of translocation of EHEC across human M cells in *in vitro* human organ culture simulated conditions, but *lpf* deletion mutant strains of *E*. *coli* O157:H7 did not show any significant effects on adherence to Caco-2 cells 3 h post-infection [12]. *E*. *coli* Common Pilus (ECP) is a fimbrial adhesin associated with biofilm formation and persistent colonization of the bovine intestine [13, 14]. Deletion mutations to the *ecp* gene reduced adherence to Hep2 cells at up to 6 h post-infection, but no similar evidence has been shown in Caco-2 or other human intestinal cells [15, 16]. The Hemorrhagic Coli Pilus (HCP) has also been shown to contribute to adherence to human and bovine cells, and also to porcine and bovine intestinal explants [17]. An *hcpA* deletion in *E*. *coli* O157:H7 reduced adherence to the human intestinal epithelial cell line HT-29 *in vitro* after 6 h post-infection, and over-expression of *hcpABC* genes also resulted in increased cellular invasion, hemagglutination, biofilm formation, and motility [18]. Flagella (H antigens) are well characterized for their role in motility but have also been implicated in adherence. Flagella in *E*. *coli* O157:H7 were able to bind bovine intestinal mucus, while H-negative mutant strains showed reduced adherence to bovine intestinal tissue explants; though other studies have reported H-negative strains as being able to colonize young calves [9, 19]. Additionally, the ETEC protein EtpA, which mimics the major subunit protein flagellin, demonstrates adherence to intestinal cells *in vitro* through the use of recombinant protein (rEtpA) in adherence assays [20]. The exact role of flagella in EHEC initial adherence to human IECs remains unknown [21]. Many other proteins have been identified as being of interest in the study of adherence, but roles in initial adherence are either unclear, not studied in human cells, or do not yet have enough data to be conclusive [7].

A unique adherence mechanism has been described in the human mucosal pathogen *Streptococcus pneumoniae*, involving the human polymeric immunoglobulin receptor (pIgR). Under normal conditions, the pIgR system plays a critical role in innate mucosal immunity [22]. The human pIgR is a glycoprotein varying in size from 80–120 kDa depending on the level of glycosylation, and primarily functions as a transport for dimeric immunoglobulin A (dIgA) into the intestinal lumen [23]. In the lumen, the transported pIgR-dIgA complex is cleaved from the IEC surface and termed secretory IgA (sIgA), where it plays an important role in maintaining the balance between immune function and microflora homeostasis [24, 25]. In addition, pIgR lacking dIgA can also be transported. The unbound pIgR is also cleaved and released (free secretory component or SC), which also serves as part of innate immunity [26, 27]. Once sIgA or SC has been secreted into the intestinal lumen, a retrograde transport pathway is used to recycle the membrane-bound pIgR [28]. *S*. *pneumoniae* uses a choline-binding protein PspC as an adhesin, binding the pIgR, SC, or sIgA at domains D3 and D4 [22, 29]. PspC enhances adherence and invasion of nasopharyngeal epithelial cells through use of the pIgR recycling transport pathway [28, 30]. Although *S*. *pneumoniae* is a Gram-positive pathogen, it is a pathogen that targets mucosal epithelial cells, as does EHEC [29, 31, 32]. High expression of pIgR in the intestine presents an opportunity for other pathogens to utilize a similar mechanism for adherence [24]. A separate study in 2007 found that *pigR* expression was upregulated in bovine hosts when experimentally infected with *E*. *coli* O157:H7 and a non-O157 EHEC strain for 6 h (1.93 and 3.51 fold-change (FC), respectively), but not in response to non-colonizing control strains [33]. Although the cause of this behavior was not determined, the results support the question of whether a similar mucosal epithelium colonizing pathogen, such as EHEC also has the potential to use pIgR for adherence during infection.

In this study, we describe the interaction between the *E*. *coli* O157:H7 outer membrane protein Slp (carbon starvation-inducible lipoprotein) and the pIgR, and its role in initial adherence to Caco-2 cells *in vitro*. Utilizing immunofluorescent microscopy, we demonstrated that during initial adherence, *E*. *coli* O157:H7 had a significant correlation with its location and the location of pIgR protein on the Caco-2 cell surface. The colocalization of *E*. *coli* cells with pIgR protein was statistically significant with *E*. *coli* O157:H7 (O157-WT), but not with the nonpathogenic *E*. *coli* strain K12. Additionally, several other non-O157 pathogenic *E*. *coli* strains also showed significant colocalization patterns. These observations were further investigated using a commercially available recombinant Fc-tagged human pIgR protein (pIgR-Fc) in a co-immunoprecipitation (Co-IP) assay, leading to the identification of Slp as the interacting protein involved. A deletion mutation of *E*. *coli* O157:H7 lacking the *slp* gene (O157-Δ*slp*) resulted in an elimination of the colocalization patterns observed with immunofluorescent microscopy, the absence of the band corresponding to Slp following Co-IP, and a significant adherence deficiency to Caco-2 cells. Furthermore, the O157-Δ*slp* strain was complemented *in trans* with the *slp* gene (O157Δ*slp*-p:*slp*), which restored the wild-type phenotypes seen in colocalization, Co-IP, and adherence to Caco-2 cells *in vitro*. Taken together, these results support the proposal of direct interaction between the *E*. *coli* O157:H7 Slp and the host pIgR and its contribution to initial adherence.

## Materials and methods

### Bacterial and mammalian cell growth and culture

All bacterial strains and plasmids are described in Table 1.

**Table 1:** Bacterial strains and plasmids.

Strains or Plasmids	Description	Genotype
**Strains**		
MG1655	*E*. *coli* K12 nonpathogenic	O Serogroup: N/A Pathotype: N/A Shiga toxin 1 (*stx1*): - Shiga toxin 2 (*stx2*): - Intimin (*eae*): - *slp*: +
O157-WT EDL 932 (ATCC #43894)	*E*. *coli* O157:H7 wild-type	O Serogroup: O157 Pathotype: EHEC Shiga toxin 1 (*stx1*): + Shiga toxin 2 (*stx2*): + Intimin (*eae*): + *slp*: +
O157-Δ*slp*	*E*. *coli* O157:H7 Δ*slp*	O Serogroup: O157 Pathotype: EHEC Shiga toxin 1 (*stx1*): + Shiga toxin 2 (*stx2*): + Intimin (*eae*): + *slp*: -
O157Δ*slp*-p:*slp*	*E*. *coli* O157:H7 Δ*slp* + puc18::*slp*	O Serogroup: O157 Pathotype: EHEC Shiga toxin 1 (*stx1*): + Shiga toxin 2 (*stx2*): + Intimin (*eae*): + *slp*: +
O26	*E*. *coli* O26 field isolate*	O Serogroup: O26 Pathotype: EHEC Shiga toxin 1 (*stx1*): + Shiga toxin 2 (*stx2*): + Intimin (*eae*): + *slp*: +
O103	*E*. *coli* O103 field isolate*	O Serogroup: O103 Pathotype: STEC Shiga toxin 1 (*stx1*): + Shiga toxin 2 (*stx2*): + Intimin (*eae*): - *slp*: +
O145	*E*. *coli* O145 field isolate*	O Serogroup: O145 Pathotype: EPEC Shiga toxin 1 (*stx1*): - Shiga toxin 2 (*stx2*): - Intimin (*eae*): + *slp*: +
**Plasmids**		
pKD119	λ Red recombinase expression plasmid	Antibiotic resistance: Tetracycline (Tet^R^) Growth: 30°C
pKD3	λ Red recombinase plasmid containing FRT-flanked chloramphenicol resistance cassette	Antibiotic resistance: Chloramphenicol (Cm^R^)
pCP20	λ Red recombinase FLP expression plasmid	Antibiotic resistance: Ampicillin (Amp^R^) Growth: 30°C
pUC18	Complementation cloning plasmid	Antibiotic resistance: Ampicillin (Amp^R^)

*Isolates were provided by Dr. Chitrita DebRoy at the *E*. *coli* Reference Center at the Pennsylvania State University, University Park, PA.

All bacterial cultures were inoculated from single colonies into Luria Bertani (LB) broth with appropriate antibiotics when applicable and grown shaking at 37°C with 5% CO_2_ overnight. Caco-2 cells (American Type Culture Collection (ATCC) HTB-37) were grown at 37°C with 5% CO_2_, in Eagle's Minimum Essential Medium (EMEM) (ATCC) plus 20% Fetal Bovine Serum (FBS) (Atlanta Biologicals) unless otherwise indicated. Caco-2 cells were seeded into tissue culture treated six well plates (Corning Life Sciences), containing glass cover slips if used for microscopy and grown to confluency.

### Immunofluorescent microscopy

Mammalian cells were grown as described, rinsed once with sterile phosphate buffered saline (PBS) and fresh medium applied 2 to 4 h prior to infection. Bacterial strains were inoculated at an MOI of 20 from overnight cultures as described under ‘Bacterial and mammalian cell growth and culture’, allowed to equilibrate to 37°C for 5 min, and incubated for 0 to 6 h. After incubation, cell culture media was removed, and cells were washed gently (by tilting and swirling) with sterile PBS three times to remove unadhered bacteria. After infection, samples were fixed using 10% neutral buffered formalin (Azer Scientific) for 10 min at room temperature and rinsed with PBS three times. Fluorescent stains were applied as listed below, with two PBS rinses in between each stain; slides were stored in glycerol-based mounting medium and away from light. The pIgR protein was stained using an unconjugated PIGR rabbit IgG polyclonal antibody (Thermo Fisher Scientific) at a 1:750 dilution in PBS for 60 min, followed by a DyLight 488-conjugated secondary goat anti-Rabbit IgG (H+L) antibody (Thermo Fisher Scientific), stained at a 1:1,000 dilution in PBS for 60 min. *E*. *coli* were stained using an unconjugated *E*. *coli* goat IgG polyclonal antibody (Thermo Fisher Scientific), stained in 1:1,000 dilution for 60 min, followed by a DyLight 594-conjugated secondary donkey anti-goat IgG (H+L) Secondary Antibody (Thermo Fisher Scientific), stained at 1:1,000 dilution in PBS for 60 min. Caco-2 cell nuclei were stained using Hoechst 33342 (Thermo Fisher Scientific), stained at a 1:1,000 dilution in PBS for 5 min. Slides were washed three times in sterile PBS and mounted using glycerol based mounting media and stored away from light. Immunofluorescent images were obtained using a Keyence BZ-9000 Fluorescence Microscope (Keyence) with 40X objective lenses (Nikon) with filters for DAPI, GFP, Texas-Red, and phase contrast. Images were taken as z-stacks at 0.5 μM intervals. Covariance (the quantification of pixels with fluorescent signals in both GFP and Texas-Red channels) was measured using ImageJ (U. S. National Institutes of Health) and the JaCOP plugin [34].

### Quantitative adherence

Infection of mammalian cells was done as described. After infection and incubation, samples were collected dispensing 100 μL sterile 10% triton X-100 (Sigma-Aldrich) in PBS and incubated at room temperature for 10 min until the cells detached from the well plate. A 900 μL volume of sterile PBS was added to rinse the well, and samples were collected using a sterile cell scraper for a total sample volume of 1 mL. Samples were resuspended and vortexed vigorously until no visible cell clumps remained, ten-fold serial dilutions were made by adding 100 μL of sample into 900 μL sterile PBS in succession. A 100-μL volume of each dilution was plated and grown on LB plates overnight at 37°C before counting. All adherence assays were normalized by calculating colony forming units (CFU) per inoculum to ensure accuracy, and they were performed in triplicate. To calculate adherence, bacterial counts were adjusted by dilution factor, averaged, and the percent adherence (to normalize across samples and experiments) was calculated from the number of adhered bacteria per the number of inoculated bacteria per well. The percent adherence per well was then compared to the maximum adherence count (wild-type count at 3 h post-infection) to calculate relative adherence per sample.

$${\left(\#A)=(\#CFU)\times 10\right)}^{\left(PDF\right)}$$

(%AI)=[(#A)/(#1)]×100

(%RA)=(%AI)/(%AM)

Where (#A) = number of adhered bacteria; (#CFU) = number of colonies counted per plate; (PDF) = plate dilution factor; (#I) = number of inoculated bacteria per well (sample); (%AI) = percent adherence of inoculum (normalization between samples); (%AM) = maximum percent adherence (%AI) of wild-type bacteria (3-h samples); (%RA) = relative percent adherence (values shown in figures). P-values were calculated using the mean %RA for the Student’s T-test and p ≤ 0.05 was considered significant [35].

### Quantitative PCR (qPCR)

Quantitative real-time polymerase chain reaction (qPCR) was done using mRNA purified from bacteria adhered to eukaryotic cells as described under ‘Immunofluorescent microscopy’ (relative expression during adherence) or mRNA purified from bacteria grown in LB broth culture over time (relative expression over time). The mRNA purification was performed using the Ambion Ribopure Kit (Thermo Fisher Scientific) for total RNA recovery, Ambion MicrobEnrich Kit (Thermo Fisher Scientific) to purify prokaryotic RNA, and Ambion MicrobExpress Kit (Thermo Fisher Scientific) to purify prokaryotic mRNA. All mRNA samples were reverse-transcribed to cDNA using the iScript cDNA Synthesis kit (BioRad). The cDNA samples were then concentrated and cleaned of reverse transcription reaction components using ethanol precipitation: to each sample, 10% (of total reverse transcription reaction volume) volume of 3 M sodium acetate pH 5.2 was added; 2.5 volumes of 100% ethanol was added; sample was vortexed thoroughly and incubated at -20°C for a minimum of 6 h to form a DNA precipitate. After precipitation, samples were centrifuged (in a standard microcentrifuge) at ˃10,000xg for 30 min at 4°C; pellets were rinsed once with ice-cold 70% ethanol; pellets were then air dried and resuspended in water and assessed for purity and concentration using AD 260/280 ratios. Using cDNA as PCR template, 20 μL qPCR reactions were made using 50–500 ng of template cDNA, 10 μL Applied Biosystems SYBR Green PCR Master Mix (Thermo Fisher Scientific), and molecular-grade water up to 20 uL total volume. The qPCR thermal cycling was done using the 7500 Fast Real-Time PCR System (Applied Biosystems) using the default PCR cycling settings (hold stage: 10 min at 95°C; cycling stage (40 cycles): 15 sec at 95°C, and 1 min at 60°C) and the primers listed in Table 2.

**Table 2:** Primer sequences used in this study.

Gene	Reference Sequence	Purpose	Sequence 5’-3’
***eae***	NC_002695.1	qPCR	F:GTCGTGTCTGCTAAAACCGC R:CGGCGGAACTGGAAGTTAGT
***gapA***	NC_002695.1	qPCR	F:ACTTCGACAAATATGCTGGC R:CGGGATGATGTTCTGGGAA
***slp***	NC_002695.1	qPCR	F:GTTACCATCCTCGGCACCAT R:CAAATGCCACACCTGGATGC
***slp***	NC_002695.1	Mutation	F:GTGCTGCTAATGCGGATGCGACYTTCAAGGTTCAGTGTGTAGGCTGGAGCTGCTTC R:TTACTGATAGGTTAAAGAGAACCAGGCCTGTGCATTCATATGAATATCCTCCTTAG
***slp***	NC_002695.1	Screening	F:AGGTGCACTCATACTCAGCC R:TGCACCATAGCCGTAATCCC
***slp***	NC_002695.1	Sequencing	F:TCGCCTCAGAATCAGATGAAA R:ATCTGCATCTTTCGGTGGTG
***slp***	NC_002695.1	Complementation	F:TAAGCAAAGCTTATGGTTTTAATATTTGTTGATAAG R:TGCTTAGAATTCTTATTTGACCAGCTCAGGTGTTAC

Relative expression was calculated using the relative expression ratio where efficiency (E) was calculated using the slope of a standard curve of ten-fold serial dilutions of DNA template, and FC was calculated using the relative expression ratio as described by Pfaffl [36].

$${E=10}^{\left[-1/slope\right]}$$

$$FC=\frac{{\left(E_{\mathrm{target}}\right)}^{\Delta Ct_{\mathrm{target}}(control-sample)}}{{\left(E_{\mathrm{ref}}\right)}^{\Delta Ct_{\mathrm{ref}}(control-sample)}}$$

when E = efficiency of target or reference gene, ΔCt = (Ct value of control-Ct value of sample), target = the gene of interest, ref = endogenous control gene, control = untreated sample, and sample = experimental sample [36]. A FC ≥ 2 was considered significant.

### Gene deletion and complementation

*E*. *coli* O157:H7 was used to make genomic deletions using the lambda Red recombinase system described by Datsenko and Wanner [37]. Bacterial strains hosting plasmids were grown with antibiotics according to their respective resistance genes, and plasmids were purified using the Plasmid Midi Kit (Qiagen). PCR, using the mutation primers listed in Table 2, was performed using plasmid pKD3 as a template on an Eppendorf MasterCycler PCR System (Eppendorf). PCR reactions were done in 50 μL volumes: 0.25 μL Taq DNA Polymerase enzyme (5 U/μL) (Omega Bio-Tek); 10X PCR buffer (#TQ2100-00, Omega Bio-Tek) at 5 μL; 1.0 μL dNTPs (10mM) (Lucigen); 1.0 μL each of forward and reverse primers (20 μM) (IDT), and water to 50 μL total volume. All PCR cycling conditions were done as follows: 95°C for 5 min; 35 cycles of 95°C for 5 min, 55°C for 30 sec, 72°C for 3 min; followed by a final extension step of 72°C for 5 min. PCR products were run in a 1.5% agarose gel in Tris-acetate-EDTA (TAE) buffer at 150 V for 1 to 2 h to obtain clear band resolution, bands were excised from the gel, and purified using the MinElute Gel Extraction Kit (Qiagen). PCR products were transformed into electrocompetent *E*. *coli* O157:H7 containing plasmid pKD119, using volumes between 2 and 5 μL at concentrations between 100 and 500 ng/μL (as measured by Nanovue spectrophotometer). Transformed cells were incubated in 1 mL of pre-warmed 37°C SOC medium and incubated at 37°C for 1 h, and then plated on LB containing chloramphenicol. Colonies were selected for antibiotic resistance and screened by PCR. Transformants positive for antibiotic resistance and negative for screening PCR products were grown and made electrocompetent, transformed with plasmid pCP20 to excise the antibiotic resistance cassette, and screened for antibiotic resistance. Deletions were confirmed by sequencing the junction sequences flanking the deletion site, using The Pennsylvania State University’s Genomics Core Facility (University Park, PA). Plasmid complementations of deletion strains were made using pUC18 and the primers listed in Table 2. PCR was done using *E*. *coli* O157:H7 genomic DNA as a template; and PCR reactions, PCR cycling conditions, PCR product purification, and transformations were done as described.

### Co-immunoprecipitation (Co-IP) and protein identification

The Co-IP assay was done using the Invitrogen Dynabeads Protein A Immunoprecipitation kit (Invitrogen). Bacterial cultures were grown from starter culture diluted into 100 mL LB broth at an OD of 0.05 and grown with shaking to prevent sediment formation for approximately 14 h at 37°C. Bacterial pellets were collected by centrifugation at 4°C at 4,000xg for 20 min and resuspended in 5mL water for sonication. Sonication was done using a hand-held sonicator (Thermo Fisher Scientific) on ice for a total of 20 min per sample, or until lysate turbidity was visibly reduced. A 5 mL volume of sonicated bacterial lysate was aliquoted into 1 mL samples. Untreated lysate was not incubated with protein; treated lysate was incubated with 10 μg of Recombinant Human Polymeric Immunoglobulin Receptor/PIgR (C-Fc) CI09 protein (Novoprotein) containing an Fc tag at the C-terminus of the protein. Protein-lysate incubation was done overnight at 4°C for 16–18 h on a rotator, and proteins collected using the Co-IP kit. Protein A coated beads from the kit were prepared for use and incubated with the lysate for 30 min at 4°C on a rotator. Beads were collected using a magnet stand and washed twice with kit wash solution. Beads pellet was then resuspended in 10 μL of kit elution solution and 10 μL of sodium dodecyl sulfate polyacrylamide gel electrophoresis (SDS-PAGE) buffer and incubated at 95°C for 5 min before loading into a 12% acrylamide SDS-PAGE gel. The SDS-PAGE gel was run for 2 h at 100 V at room temperature and stained with Coomasie blue (BioRad). All protein identifications were done at protein facilities using liquid chromatography and tandem mass spectrometry (LC MS/MS) analysis. A total of three analyses were completed, at The Pennsylvania State Proteomics and Mass Spectrometry Core Facility (1) and the Protein Facility of the Iowa State University Office of Biotechnology (2 and 3). The protein bands were cut from the SDS-PAGE gel and processed at the respective facility. Peptide fragmentation patterns were compared to known databases of proteins of *E*. *coli* (PSU using SEQUEST and Uniprot; ISU using Mascot or Sequest HT). Raw data were analyzed using Thermo Scientific's Proteome Discoverer Software.

### Statistical analysis

All quantitative assays were performed in triplicate in independent experiments. Data are shown as the mean values ± standard deviation (SD) of the mean. P values were calculated using the two sample Student’s t test. Threshold for significance was p < 0.05 [35]. Analysis was done using XLSTAT 2018 (Addinsoft).

## Results

### Initial adherence timeline of *E*. *coli* O157:H7 during adherence to Caco-2 cells

**Fig 1:**
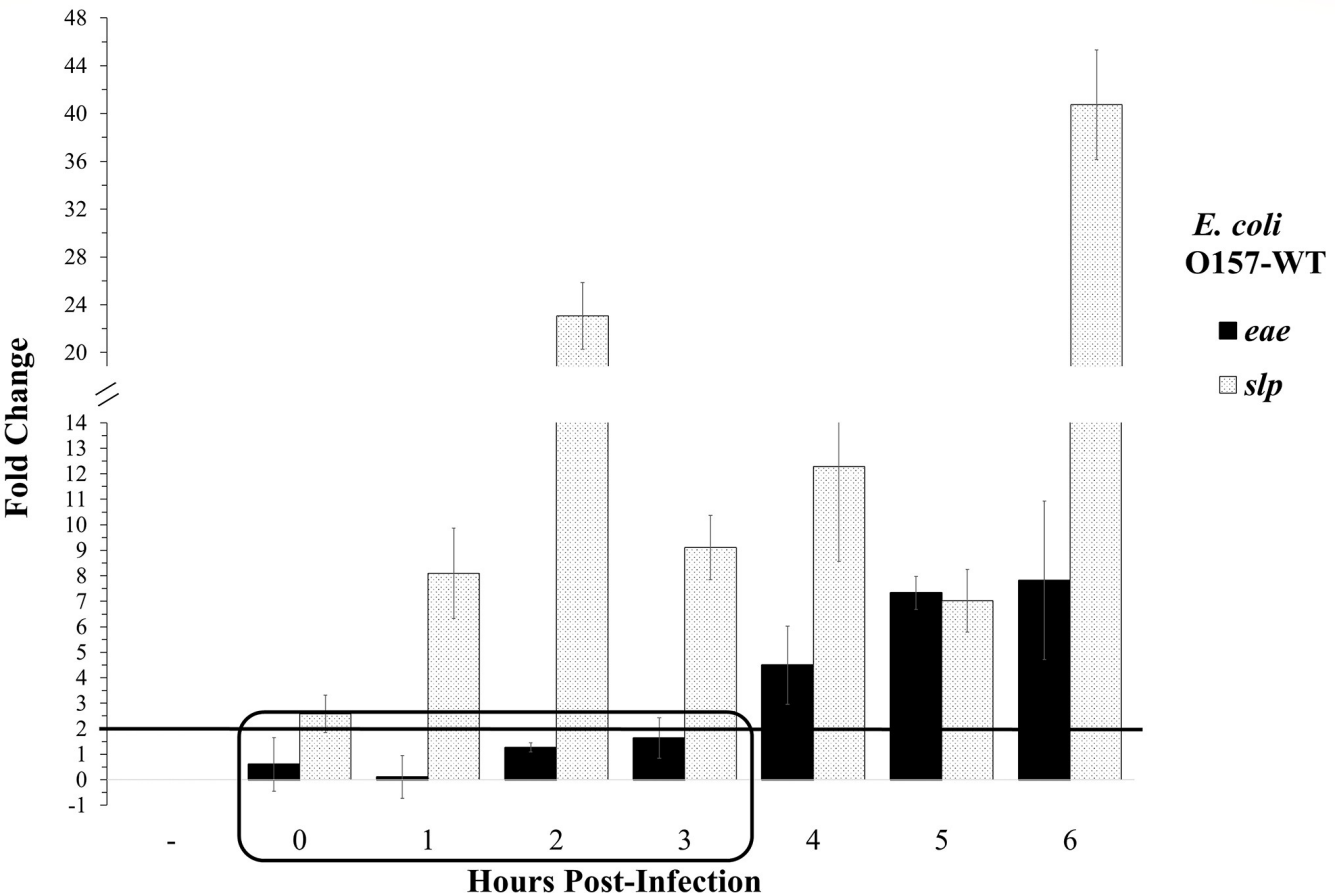
Relative expression of *eae* and *slp* during *E*. *coli* O157:H7 adherence to Caco-2 cells and the initial adherence timeline of *E*. *coli* O157:H7 *in vitro*. Relative expression of *eae* and *slp* during adherence are shown as the fold-change (FC) over 6 h post-infection, as compared to *E*. *coli* O157:H7 grown in cell culture mediun alone (control). Significant upregulation is defined as FC ≥ 2.0. Threshold for significance is shown at FC = 2.0, and initial adherence timeline is shown in the boxed area.

In order to effectively study the roles of different factors during attachment, a specific timeline was required to define initial adherence in this model. Using the expression of LEE-encoded genes as a benchmark for the beginning of the intimate adherence stage of attachment, significant LEE upregulation has been cited as taking place between two and 6 h post-infection *in vitro*. When adhered to Caco-2 cells, *E*. *coli* O157:H7 Sakai demonstrated increased production of EspA, EspB, and Tir protein production after 4.5 h post-infection [38]. During adherence to HeLa cells, LEE1 in *E*. *coli* O157:H7 Sakai has been shown to be activated by 4 h post-infection, and LEE1 and LEE5 demonstrated transient upregulation when exposed to an embryonic bovine lung cell line [39, 40, 41]. Expression of *eae* in an undefined *E*. *coli* O157:H7 strain demonstrated upregulation as early as 2 h after infection of HeLa cells [42]. Other studies of EPEC have shown LEE promoter or LEE-encoded gene upregulation between 4 and 6 h in several different epithelial cell lines, but the differences in the LEE operon between EPEC and EHEC strains make the relevance to *E*. *coli* O157:H7 unclear [8, 43]. Following previous work, under the specific conditions of this study the intimin-encoding *eae* gene was used as a benchmark for the onset of intimate adherence in this model. To study the gene expression profiles of initially attached bacteria only, the FC of *eae* expression in adhered *E*. *coli* O157:H7 in comparison to a non-adhered control culture was measured by qPCR using the relative expression ratio method for comparative Ct analysis [36]. The expression of *eae* surpassed the threshold for significance (FC ≥ 2) after 4 h and increased thereafter, consistent with previous literature (Fig 1). Thus, initial adherence in this study was defined as taking place from 0–3 h post-infection, when *eae* was not significantly upregulated. Additionally, the expression of *slp* was significantly upregulated at all time points as compared to *E*. *coli* O157:H7 grown in cell culture media alone at the same time points shown.

### Colocalization of pIgR with *E*. *coli*

**Fig 2:**
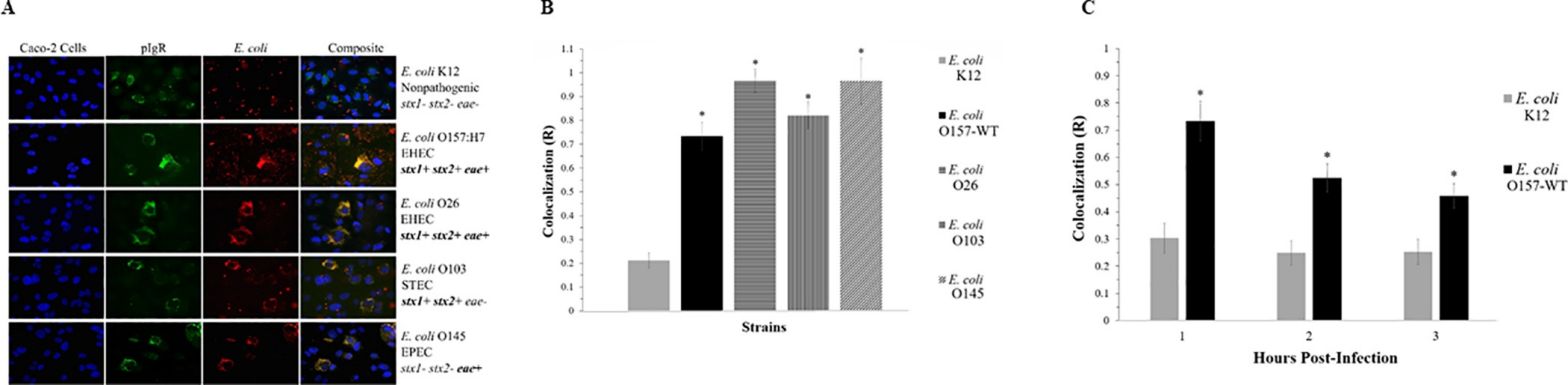
Colocalization of pIgR with *E*. *coli* strains during initial adherence. (A) Colocalization of pIgR with *E*. *coli* strains at 2 h post-infection. Fluorescent signals of Caco-2 nuclei, Hoechst stain with emission at 497 nm, shown in blue; fluorescent signals of *E*. *coli*, DyLight 594 with emission at 594 nm, shown in red; fluorescent signals of pIgR protein, DyLight 488 with emission at 488 nm, shown in green; colocalization of pIgR protein with *E*. *coli* K12 and O157-WT (B) Colocalization of pIgR with *E*. *coli* strains, and (C) pIgR with *E*. *coli* K12 and O157-WT over time. Statistical significance, shown as *, was as compared to the *E*. *coli* K12 control at the same time points. *P*-values < 0.05 were considered significant.

To investigate the possibility of a pIgR-mediated initial adherence mechanism in *E*. *coli* O157:H7, immunofluorescence was used to assess colocalization between the pIgR protein and adhered *E*. *coli* bacteria and quantify any correlation using covariance analysis (Fig 2A). The colocalization, shown as R, attested that there was no significant correlation between the locations of *E*. *coli* K12 bacteria and the pIgR protein; while O157-WT did exhibit a significant correlation (Fig 2B and 2C). This relationship is further detailed when observed over time, where O157-WT showed an initial peak of colocalization (R > 0.7) at one hour post-infection, and decreased over 2 and 3 h (R > 0.5 and 0.4, respectively). This pattern of early peak followed by steady decrease was consistent with the predicted pattern of an adhesin active during initial adherence, as its activity would be less necessary as the intimate adherence factors assumed adherence function at later time points. No such pattern was observed in *E*. *coli* K12. Several other non-O157 intestinal pathogenic strains of *E*. *coli* were also tested and showed significant colocalization at 2 h post-infection, indicating the possibility that an adherence mechanism is not limited to only *E*. *coli* O157:H7 (Fig 2A and 2B).

### Direct interaction between *E*. *coli* O157:H7 and the pIgR

**Fig 3:**
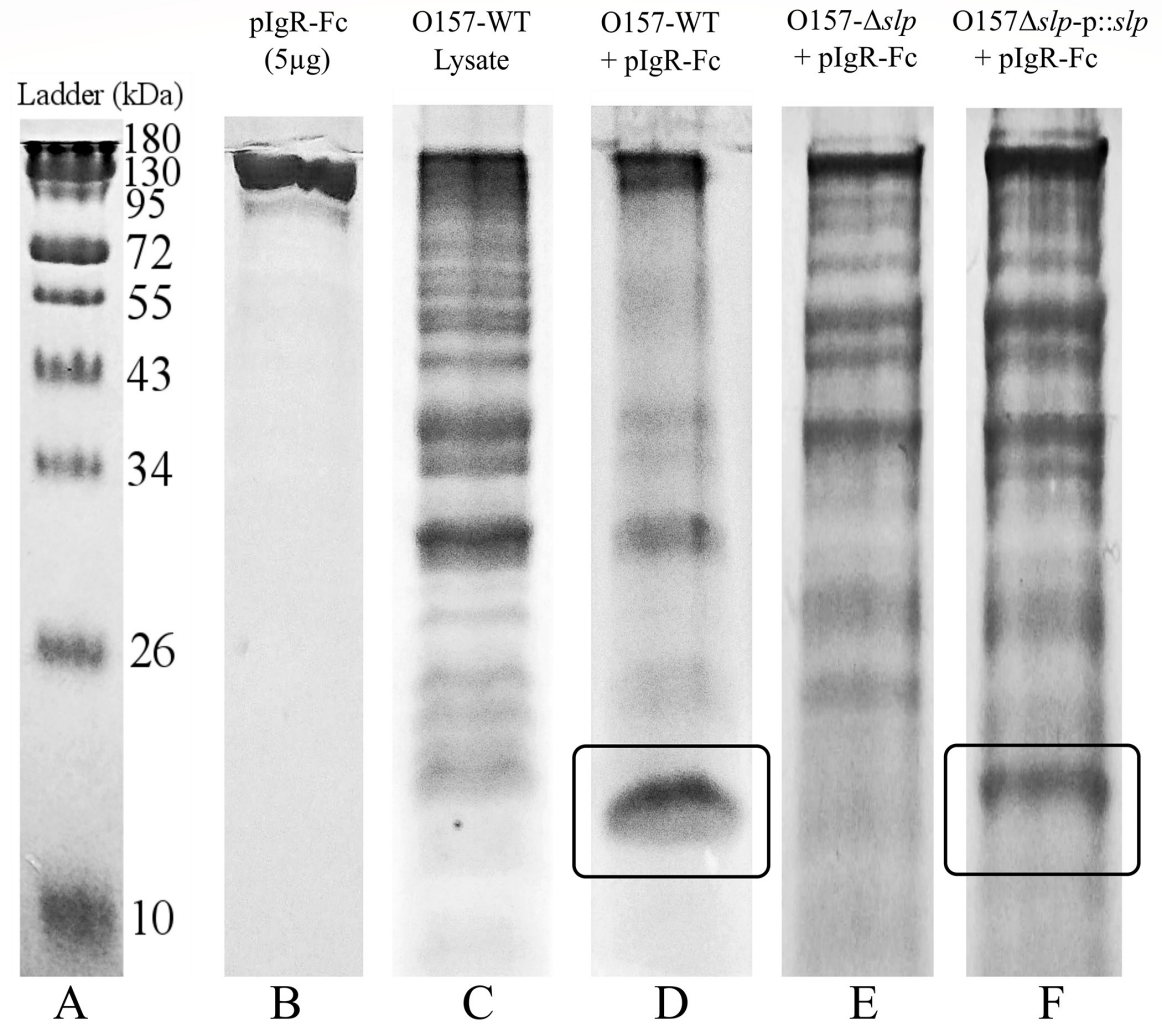
Co-immunoprecipitation of *E*. *coli* O157:H7 proteins with human recombinant Fc-tagged pIgR protein. SDS-PAGE showing the apparent sizes of proteins in the conditions used for Co-IP. The protein of ~20 kDa in size, which was co-precipitated with pIgR is enclosed in a box. The bands in the boxed areas were selected for LC MS/MS analysis.

To demonstrate a direct interaction between *E*. *coli* O157:H7 and the pIgR, Co-IP assay was done using a C-terminal Fc tagged human recombinant pIgR protein (pIgR-Fc) and O157-WT proteins (whole-cell lysate was used to avoid the accidental exclusion of any proteins). Recovered proteins were run in a reducing SDS-PAGE gel (Fig 3), and bands of interest (shown in boxes) were later identified through LC MS/MS analysis (Table 3). This experiment and analysis were repeated three times, and Slp was identified in all three analyses with the highest sequence coverage. Lane D shows the presence of a concentrated band of approximately 20 kDa (enclosed in a box) in the treated sample, not visible in the untreated control sample (Lane C), indicating the likelihood of a direct binding of that bacterial protein with the pIgR-Fc (Fig 3). There were several other observable bands in the treated sample between 26–43 kDa, but they corresponded in size to the most concentrated bands present in the control, making it likely that they were non-specific and not indicative of protein binding. A direct relationship between the O157-WT protein Slp and the pIgR-Fc protein was demonstrated through disruption of the *slp* gene. A deletion mutation of the *slp* gene (O157-Δ*slp*) eliminated the band of interest recovered with the wild-type strain (O157-WT) (Lane E), and the subsequent complementation of a plasmid-encoded *slp* gene (O157Δ*slp-p*:*slp*) restored the wild-type phenotype (Lane F) (Fig 3).

**Table 3:** Protein identification using LC MS/MS. Summary of the outer membrane proteins found to be possible identifications of the protein recovered from Co-IP. Proteins are listed in order of decreasing sequence coverage, and proteins that were identified in three of three LC MS/MS analyses are noted.

Protein ID	Protein Description	Reference Sequence and Accession	Average % Coverage	# AAs	kDa
*Slp	Outer membrane protein Slp	*Escherichia coli* O157:H7 Str. EDL933 AAG58638.1	54.9	199	22.2
*Pal	Peptidoglycan-associated lipoprotein	Multispecies WP_001295306.1	53.8	173	18.8
*OmpW	Outer membrane protein W	Multispecies WP_000737224.1	49.8	212	22.9
*OmpX	Outer membrane protein X	Multispecies WP_001295296.1	44.7	171	18.6
OmpA	Outer membrane protein A precursor	*Escherichia coli* O157:H7 Str. EDL933 AIG67419.1	22.2	354	38.1
OmpC	Outer membrane protein C precursor	*Escherichia coli* WP_000865552.1	15.4	367	40.3

* Proteins present in all three LC MS/MS analyses.

### Slp expression over time and initial adherence *in vitro*

**Fig 4:**
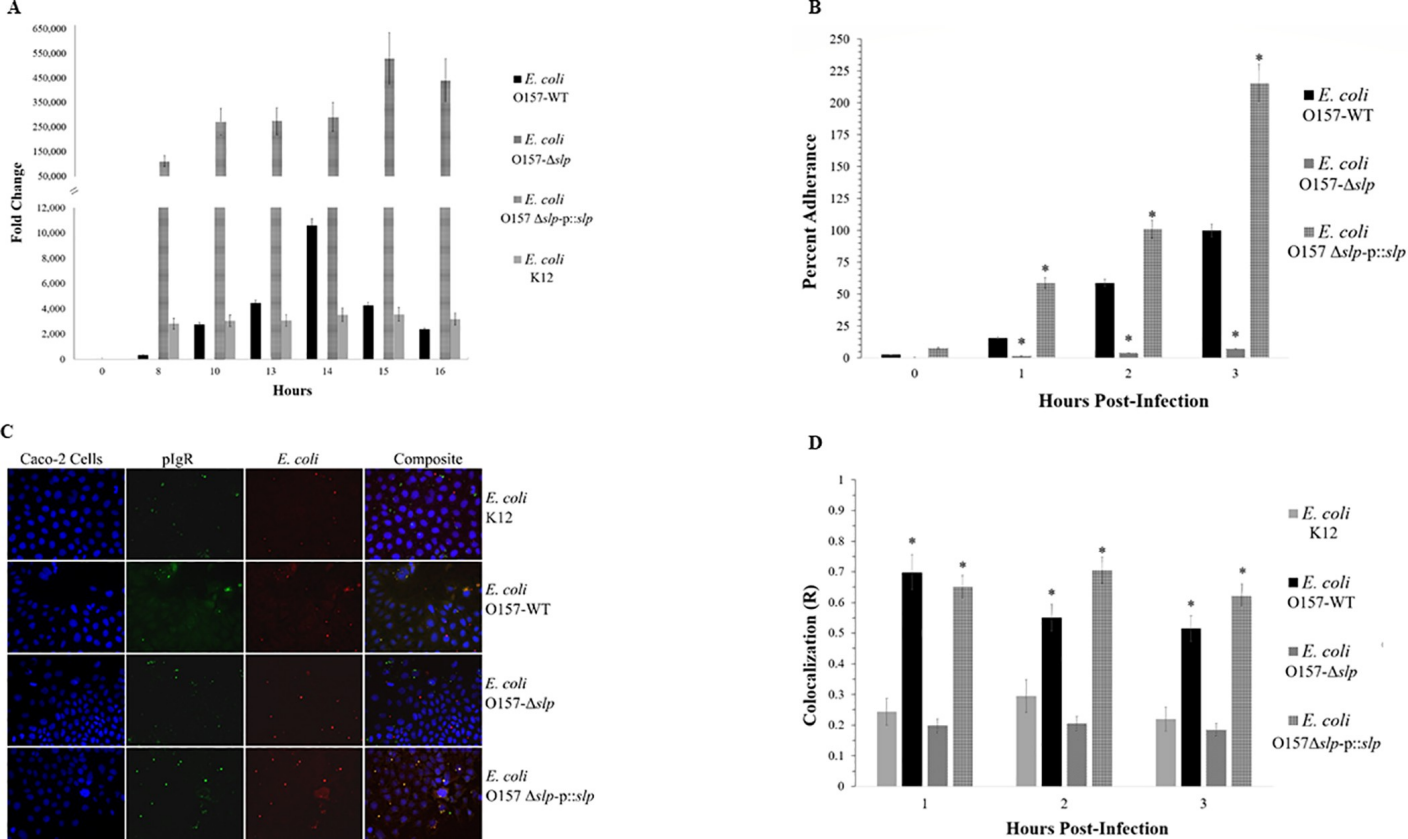
Relative *slp* gene expression, colocalization, and adherence in *E*. *coli* strains. (A) Relative gene expression of *slp* over time in LB broth culture as measured by qPCR, when compared to expression at time 0. (B) Quantitative adherence of *E*. *coli* strains to Caco-2 cells over time, relative to the maximum number of adhered wild-type bacteria (100% adherence is wild-type at 3 h post-infection). Statistical significance, shown as *, is as compared to the O157-WT strain at the same time points. (C) Fluorescence and (D) colocalization of pIgR with *E*. *coli* O157-WT, O157-Δ*slp*, and O157Δ*slp*-p:*slp*. Statistical significance, shown as *, is as compared to the *E*. *coli* K12 control at the same time points. *P*-values < 0.05 were considered significant.

Relative expression of the *slp* gene showed differing patterns between *E*. *coli* K12, O157-WT, O157-Δ*slp*, and O157Δ*slp*-p:*slp* in LB broth culture over 16 h of growth, as measured by qPCR (Fig 4A). The *slp* gene is reported to be transcribed at a low baseline level in most conditions and upregulated during entry of bacteria into stationary-phase [44]. The O157-WT *slp* expression showed a transient peak at 14 h corresponding to the entry of the culture into stationary-phase growth. *E*. *coli* K12 *slp* expression remained relatively stable and low throughout growth, and did not show any peaks or changes at any time point. The O157-Δ*slp* strain did not show any detectable levels of gene expression, while the O157Δ*slp*-p:*slp* strain showed high levels of constitutive over-expression of *slp* at high levels at all time points, ranging from 10 to over 100 magnitude differences in FC as compared to *E*. *coli* K12 and O157-WT.

When measuring initial adherence to Caco-2 cells by quantifying the number of attached bacteria over time, the O157-WT presented a pattern showing a steady increase of adhered bacterial cells over 3 h (Fig 4B). These results were consistent with O157-WT bacterial cells as they were steadily coming into contact with and attaching to the Caco-2 cells over time, as the adherence increased two- to three-fold per hour (approximately 20%, 55%, and 100% at 1, 2, and 3 h post-infection, respectively). When compared to the wild-type, deletion of the *slp* gene resulted in a significant adherence deficiency to Caco-2 cells, with O157-Δ*slp* adherence not reaching more than 10% at any time point. The O157-Δ*slp* adherence increased very slightly over 3 h (from <5% to <10% at 1 and 3 h post-infection, respectively). Conversely, the O157Δ*slp*-p:*slp* showed a hyper-adherent pattern, surpassing wild-type levels at all time points. The O157Δ*slp*-p:*slp* adherence followed roughly the same pattern as seen in the wild-type, with adherence increasing 2 to 3-fold per h (approximately 60%, 100%, and 225% at 1, 2, and 3 h post-infection, respectively). At 3 h, the number of adhered O157Δ*slp*-*p*:*slp* cells was more than double the wild-type.

Colocalization was measured in the O157-Δ*slp* and O157Δ*slp*-p:*slp* strains over 3 h of adherence to Caco-2 cells (Fig 4C and 4D). As in Fig 2, *E*. *coli* K12 did not show any statistically significant colocalization or any changes over time (R = 0.25, 0.28, and 0.22 at 1, 2, and 3 h post-infection). The O157-WT cells showed a significant colocalization at all timepoints, with the highest correlation (R = ~0.7) seen at 1 h post-infection, with a decrease to R = 0.55 and 0.50 at 2 and 3 h respectively. The O157-Δ*slp* strain demonstrated adherence levels comparable to *E*. *coli* K12, with significantly diminished colocalization and no changes over time (R = approximately 0.2 at all time points). The O157Δ*slp*-p:*slp* strain restored colocalization comparable to that of the wild-type strain, but colocalization did not decrease over time in the same manner (R = 0.65, 0.70, and 0.63 at 1, 2, and 3 h post-infection, respectively).

## Discussion

Initial adherence of EHEC bacteria is a known key step in pathogenesis [45]. Initial adherence in EHEC strains is not yet fully understood, and it is becoming clear that mechanisms are not conserved even among EHEC strains with very similar virulence profiles, and putative adhesins have inconsistent behavior between host type [7, 46–48]. In this study, we propose an *in vitro* initial adherence mechanism involving the *E*. *coli* O157:H7 outer membrane protein Slp and the pIgR protein expressed on human colonic epithelial cells.

We hypothesized that the pIgR may be involved in EHEC adherence, and determined the bacterial protein binding to the human pIgR using a Co-IP assay. The Co-IP produced a distinct band of approximately 20 kDa that was significantly more concentrated when *E*. *coli* O157:H7 proteins were incubated with pIgR-Fc than with *E*. *coli* O157:H7 proteins alone. Identification using LC MS/MS led to the *E*. *coli* O157:H7 outer membrane protein Slp, a 22 kDa lipoprotein that has not been fully characterized. Its encoding gene (*slp*) expression is primarily known to be upregulated in pure culture during entry into stationary-phase growth or during carbon starvation [44]. Its function is generally accepted to be involved with membrane stability during stationary-phase growth, but additional alternative functions have also been proposed [49–51]. There has also been evidence of increased *slp* gene expression during biofilm formation, though the significance of these findings is unclear [52]. The *slp* is located on a genomic acid fitness island (AFI) along with several other defined and undefined genes and operons [53, 54]. The *slp* gene and AFI are present in all *E*. *coli*, but there are significant differences between strains. In *E*. *coli* K12, the AFI is approximately 14 kb, whereas the *E*. *coli* O157:H7 AFI is 23 kb due to the insertion of an O-island sequence [54–57]. These differences in sequence, combined with differences in transcription regulation, provide insight into possible reasons why *E*. *coli* K12 does not show a colocalization phenotype with pIgR despite having the *slp* gene. As shown in Fig 4A, the *E*. *coli* K12 *slp* gene is not expressed under the same culture conditions as *E*. *coli* O157:H7, suggesting that *slp* expression is managed by a different mechanism in *E*. *coli* K12 than in *E*. *coli* O157:H7 [54].

It is notable that Slp contributes to initial adherence and is located on an acid fitness island, because pH is a potent signaling factor for virulence and adherence gene expression, and the acidic pH encountered by *E*. *coli* in the stomach results in a gene expression profile fit for initial adherence [14]. Acid resistance response regulators downregulate the expression of LEE-encoded effectors not required during the early phases of attachment (i.e. *eae* and other intimate adherence related genes), and upregulate genes related to motility and initial adherence [14, 55, 58, 59]. Given that one of the primary acid resistance response transcription regulators GadE is known to be highly involved in the regulation of AFI gene expression where *slp* is located, it follows that pH dependent regulation may play a key role in expression of initial adhesins [56, 59]. Given the complexity and sensitivity of acid-responsive regulatory mechanisms, further study is required to fully understand these systems and how their gene products, such as Slp, affect pathogenesis *in vivo*.

The role of the pIgR in pathogenesis is a known mechanism for other pathogens, but it had not been demonstrated in *E*. *coli* adherence. EHEC infection can affect *pigR* expression; however, the effects of EHEC on the pIgR system do not explain the results reported [60–63]. In order to upregulate *pigR* expression as early as 0 to 3 h post-infection, exposure of the host cell to immunogenic signals such as LPS or Stx would have to be immediate, prolonged and stable; which is unlikely to occur in the variable intestinal environment [61]. Additionally, there is no evidence to suggest that the pIgR has any mechanism of location specificity, and any inflammatory signals only affect the rate at which pIgR reaches the membrane without any effect on its location [24, 64, 65].

*E*. *coli* O157:H7 and other EHEC strains pose a large public health burden worldwide, and the need for effective treatments or interventions is urgent [3]. Interventions are aimed at preventing transmission from the colonized bovine host at either the pre- or post-harvest stage, by treating or preventing EHEC colonization before slaughter, or reducing or eliminating contamination post-harvest [66]. To date, interventions such as vaccines and post-harvest sanitation have produced limited effects, but the identification of an outer membrane protein highly conserved among *E*. *coli* may provide a potential new target [66–68]. Bacterial lipoproteins are a broad range of proteins and have been known to be involved with many virulence functions, including adherence, and other genes with low levels of sequence homology have been identified in other Gram-negative bacteria or enteric pathogens, which suggests the possibility of a similar role among some limited classes of bacteria [50, 69]. In *S*. *pneumoniae*, the cell signaling pathway responsible for internalization is also used by other pathogens of diverse nature such as *Staphylococcus aureus*, *Neisseria meningitidis*, and *Listeria monocytogenes* [28], suggesting the possibility that this adherence mechanism might be more widespread than previously known [28, 70, 71].
